# Impact of offset polyethylene liners on acetabular component loosening: an Australian Orthopaedic Association National Joint Replacement Registry-based analysis

**DOI:** 10.1302/2633-1462.76.BJO-2026-0033.R1

**Published:** 2026-06-05

**Authors:** Seyyed Hossein Shafiei, Marcos Raul Latorre, Andrew Shimmin

**Affiliations:** 1 Melbourne Orthopaedic Group (MOG), Melbourne, Australia; 2 Orthopaedic Minimally Invasive Surgeries (OMIS) Research Centre, Sina Hospital, Tehran University of Medical Sciences, Tehran, Iran; 3 Department of Surgery, Monash University, School of Clinical Sciences, Melbourne, Australia

**Keywords:** Total hip arthroplasty, Lateralized liner, Offset liner, Acetabular loosening, Implant survivorship, AOANJRR, Highly cross-linked polyethylene, acetabular component loosening, polyethylene, Anesthesiologists, osteoarthritis (OA), Australian Orthopaedic Association National Joint Replacement Registry (AOANJRR), primary total hip arthroplasty, highly cross-linked polyethylene (HXLPE), posterior approach, Total hip arthroplasty (THA), hip biomechanics

## Abstract

**Aims:**

Restoring native hip biomechanics is essential for the long-term success of total hip arthroplasty (THA). Lateralized (offset) acetabular liners were developed to increase global offset and improve soft-tissue tension, but their effect on implant fixation and revision risk remains uncertain.

**Methods:**

A retrospective cohort study was conducted using data from the Australian Orthopaedic Association National Joint arthroplasty Registry (AOANJRR). All primary conventional THAs for osteoarthritis performed between 2012 and 2022 using uncemented acetabular components with highly cross-linked polyethylene (HXLPE) liners were included. Procedures were categorized as lateralized liners (LL) or standard liners (SL). The primary outcome was revision for any reason; secondary outcomes included revision for loosening and acetabular loosening. Cumulative percent revision (CPR) was estimated with the Kaplan–Meier method, and Cox proportional hazards models were used to compare groups after adjusting for age, sex, BMI, American Society of Anesthesiologists (ASA) grade, and surgical approach.

**Results:**

A total of 68,435 primary THAs were analyzed, including 1,182 LL and 67,253 SL procedures. Revision for any reason occurred in 3.21% of LL and 2.44% of SL cases. At nine-year follow-up, CPR was 4.1% (95% CI 2.8 to 6.0) for LL and 3.5% (95% CI 3.2 to 3.7) for SL. The adjusted hazard ratio for revision was 1.15 (95% CI 0.83 to 1.60, p = 0.386), indicating no significant difference between groups. Revision for acetabular loosening was rare and comparable (0.17% LL vs 0.12% SL).

**Conclusion:**

In this large registry-based study, lateralized liners did not increase the risk of revision or loosening compared with standard liners. Despite being more frequently used in younger, male patients and in association with the posterior approach, lateralized liners demonstrated equivalent mid-term survivorship, supporting their safe use to optimize hip biomechanics without compromising implant fixation.

Cite this article: *Bone Jt Open* 2026;7(6):753–758.

## Introduction

Total hip arthroplasty (THA) is a widely performed and highly successful procedure for managing end-stage hip osteoarthritis.^1^ Accurate restoration of native hip biomechanics is fundamental to achieving optimal outcomes following THA, as it has a direct impact on postoperative stability, function, and patient satisfaction.^2-7^ Among these biomechanical factors, global offset, which reflects the combined contribution of acetabular and femoral offsets, plays a crucial role in recreating normal hip mechanics.^5,8,9^ Ideally, the precise restoration of both acetabular and femoral offsets is the main objective.^10^ However, anatomical constraints of the femoral canal can restrict the choice of femoral components,^11^ making it necessary in some cases to increase the acetabular offset to achieve the desired global offset.

Offset or lateralized liners (LL) were developed to subtly alter hip biomechanics by shifting the centre of rotation laterally, thereby improving soft-tissue tension and reducing the risk of impingement.^8,12^ While this design is biomechanically appealing, concerns remain regarding its long-term effects on implant fixation and wear.^13,14^ Specifically, they may increase localized stress at the bone-implant interface, potentially contributing to higher loosening rates or mechanical failure.^13,14^ Despite their growing use, large-scale registry-based evidence evaluating the revision risk associated with these liner types remains limited.

This study, utilizing data from the Australian Orthopaedic Association National Joint Replacement Registry (AOANJRR), aims to investigate whether the use of lateralized polyethylene liners in primary THA is associated with an increased risk of revision compared with standard liners. We hypothesize that lateralized liners may be associated with higher revision rates due to increased stress concentrations at the implant-bone interface or polyethylene failure due to the presence of unsupported polyethylene that sits outside the edge of the metal acetabular shell.

## Methods

### Study design and data source

This study was a retrospective cohort analysis using data from the AOANJRR. The AOANJRR is a federally approved clinical quality registry established to define, maintain, and improve the quality of care for patients undergoing joint arthroplasty surgery across Australia. The registry has collected information on > 99% of all primary and revision joint replacement procedures performed nationally since 1999, with continuous validation against hospital separation and state health department records to ensure data completeness and accuracy.^15^ Data collection for hip arthroplasty commenced nationally in 1999, and additional variables such as American Society of Anesthesiologists (ASA) grade (2012),^16^ BMI (2015), and surgical approach (2015) were subsequently incorporated.^15^ Ethics approval was not required because the AOANJRR is approved by the Commonwealth of Australia as a Federal Quality Assurance Activity (F2022L00986) under Part VC of the Health Insurance Act 1973.

### Study population

All primary THAs performed for osteoarthritis (OA) using uncemented acetabular components with highly cross-linked polyethylene (HXLPE) liners between 2012 and 2024 were included. Two liner types were initially defined in the AOANJRR dataset: standard liner (SL), with no modification to acetabular offset or version; and LL, which increases acetabular offset.

Only the most frequently implanted acetabular systems from Stryker (USA), Corin (UK), and DePuy (Johnson & Johnson, USA) were analyzed, identified by catalogue numbers prespecified in the AOANJRR research request. Procedures with ASA grade V, non-HXLPE liners, or revision THAs as index procedures were excluded. Bilateral THAs were analyzed as independent observations.

### Variables collected

Variables extracted included demographic details (age, sex, BMI); clinical factors (ASA grade, diagnosis), and surgical factors (approach (anterior, lateral, posterior)).

Follow-up time was measured from the date of primary THA to the earliest of revision or death.

### Outcomes

The primary outcome was revision for any reason. Secondary outcomes included revision for: loosening (any component), acetabular loosening, dislocation/instability, and polyethylene wear.

Within the AOANJRR, revision is defined as the removal, arthroplasty, or addition of any device component, classified as major (acetabular, femoral) or minor (liner, or head).

If multiple causes were recorded, the primary reason for revision was assigned using the registry’s hierarchical algorithm.

### Statistical analysis

Descriptive statistics were used to summarize baseline demographic and surgical characteristics by liner type. Continuous variables were presented as mean (SD) or median (IQR), and categorical variables as frequencies and percentages. Between-group comparisons were performed using independent-samples *t*-tests for continuous data and chi-squared tests for categorical data.

Cumulative percent revision (CPR) and corresponding 95% CIs were calculated using the Kaplan-Meier method for all revision endpoints. Follow-up was restricted to ten years to allow time-matched comparison between groups.

Cox proportional hazards models were used to compare revision risk between liner types, expressed as hazard ratios (HRs) with 95% CI, using the standard liner group as reference. Models were adjusted for age group, sex, obesity status, ASA grade, and surgical approach. Statistical significance was set at p < 0.05.

## Results

A total of 68,435 primary conventional THAs for osteoarthritis were analyzed, comprising 1,182 procedures with LL and 67,253 with SL. Table I presents the baseline characteristics of the LL and SL groups.

**Table I.** Summary of characteristics of primary total conventional hip arthroplasties by liner type (lateralized compared with standard).

Variable	Lateralized liner (n = 1,182)	Standard liner (n = 67,253)
Mean follow-up, yrs (SD)	4.9 (2.8)	4.4 (2.7)
Median follow-up, yrs (IQR)	4.8 (2.6 to 7.2)	4.2 (2.1 to 6.5)
Mean age, yrs (SD)	66.9 (10.0)	68.1 (10.3)
**Age bracket, n (%)**		
< 55	134 (11.3)	6,650 (9.9)
55 to 64	335 (28.3)	16,428 (24.4)
65 to 74	436 (36.9)	25,290 (37.6)
≥ 75	277 (23.4)	18,885 (28.1)
Male, n (%)	930 (78.7)	30,726 (45.7)
**BMI category, kg/m** ^**2**^ **, n (%)**		
Normal (18.5 to 24.9)	211 (17.9)	14,715 (21.9)
Pre-obese (25.0 to 29.9)	471 (39.8)	25,027 (37.2)
Obese (≥ 30.0)	498 (42.1)	27,002 (40.1)
**ASA grade, n (%)**		
I	103 (8.7)	5,381 (8.0)
II	583 (49.3)	37,106 (55.2)
III	473 (40.0)	23,954 (35.6)
IV to V	23 (1.9)	812 (1.2)
**Fixation (femoral component), n (%)**		
Cementless	972 (82.2)	47,353 (70.4)
Cemented	210 (17.8)	19,900 (29.6)
**Surgical approach, n (%)**		
Anterior	170 (14.4)	26,888 (40.0)
Lateral	163 (13.8)	8,492 (12.6)
Posterior	849 (71.8)	31,873 (47.4)

ASA, American Society of Anesthesiologists.

There were 38 revisions among 1,182 LL cases (3.21%) and 1,641 revisions among 67,248 SL cases (2.44%). Table II summarizes reasons for revision. Revisions for loosening were observed in 0.6% of LL cases and 0.4% of SL cases. Acetabular loosening and wear were infrequent in both groups.

**Table II.** Reasons for revision by type of acetabular liner (primary diagnosis of osteoarthritis).

Liner type	Procedures, n	Revision for any reason, n (%)	Revision for loosening, n (%)	Revision for acetabular loosening, n (%)	Revision for acetabular insert wear, n (%)
Lateralized	1,182	38 (3.21)	7 (0.59)	2 (0.17)	1 (0.08)
Standard	67,248	1,641 (2.44)	288 (0.43)	84 (0.12)	1 (0.00)

Procedures with an American Society of Anesthesiologists grade of V were excluded.

**Fig. 1:**
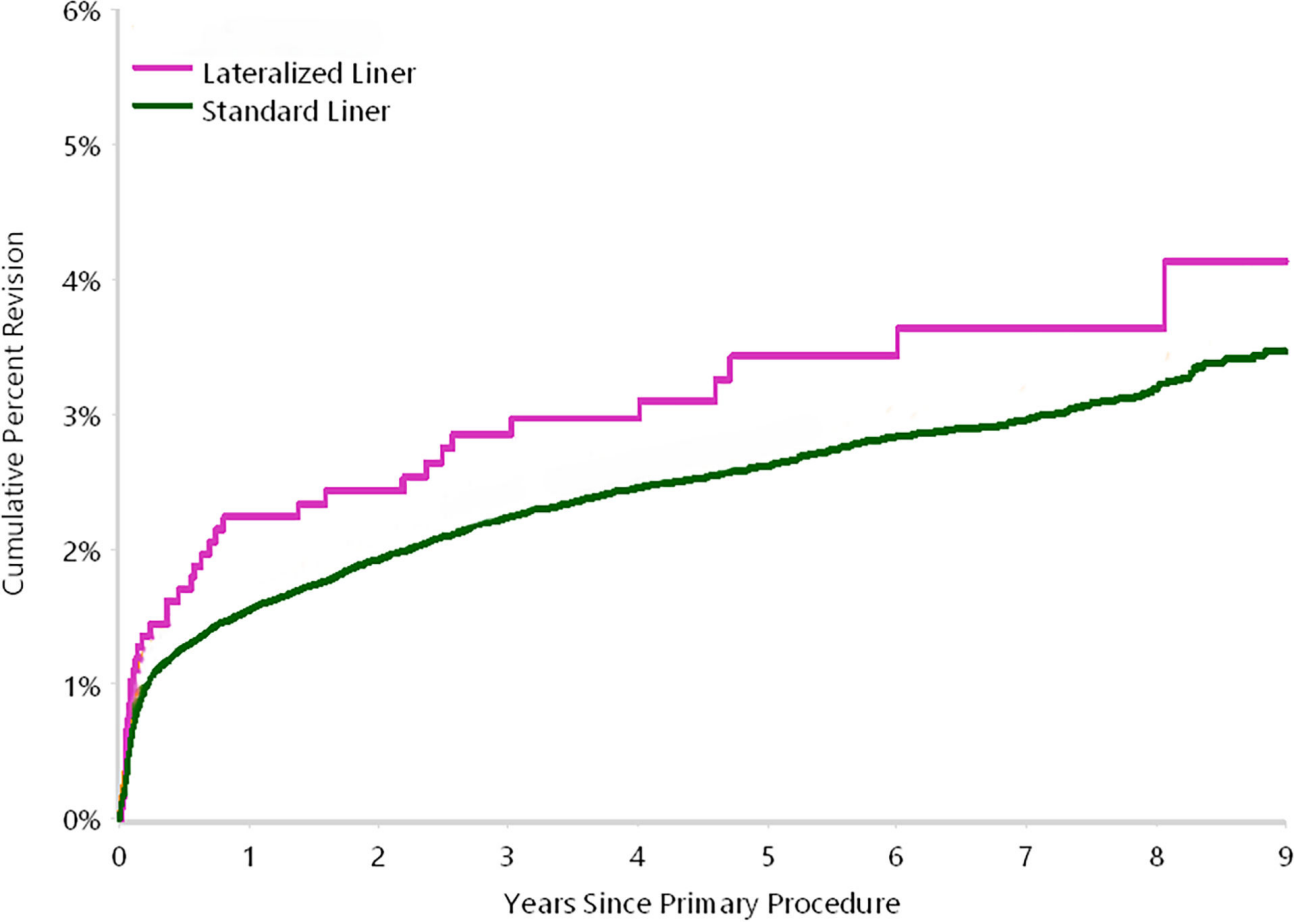
Cumulative percent revision by acetabular liner type (primary diagnosis of osteoarthritis, revision for any reason).

Figure 1 displays the yearly cumulative percent revision (CPR) for any reason, up to nine years postoperatively. LL showed slightly higher CPR estimates compared with SLs at all time points. By nine years, CPR reached 4.1% (95% CI 2.8 to 6.0) for LL compared with 3.5% (95% CI 3.2 to 3.7) for SL.

HRs for revision showed no statistically significant difference between lateralized and standard liner groups over the entire follow-up period (HR 1.15, 95% CI 0.83 to 1.60, p = 0.386). Analyses were adjusted for demographic, clinical, and surgical factors.

Table III summarizes the distribution of revision diagnoses for lateralized and standard liners within ten years postoperatively. For both groups, infection was the most frequent cause of revision, accounting for 1.4% of primaries and 42.1% of all revisions in the LL group, compared with 0.8% of primaries and 32.4% of revisions in the SL group. Loosening accounted for 0.6% of lateralized and 0.4% of standard primaries revised, with similar relative contributions to total revisions (18.4% vs 17.6%).

**Table III.** Revision diagnosis of primary conventional total hip arthroplasty by acetabular liner type (primary diagnosis of osteoarthritis, follow-up ≤ ten years).

Revision diagnosis	Lateralized, n	Primaries revised, %	Revisions, %	Standard, n	Primaries revised, %	Revisions, %
Infection	16	1.4	42.1	532	0.8	32.4
Prosthesis dislocation/instability	7	0.6	18.4	405	0.6	24.7
Fracture	3	0.3	7.9	263	0.4	16.0
Loosening	7	0.6	18.4	288	0.4	17.6
Leg length discrepancy	2	0.2	5.3	48	0.1	2.9
Pain	1	0.1	2.6	22	0.0	1.3
Malposition	1	0.1	2.6	20	0.0	1.2
Implant breakage stem	0	0.0	0.0	7	0.0	0.4
Incorrect sizing	0	0.0	0.0	7	0.0	0.4
Implant breakage acetabular insert	0	0.0	0.0	6	0.0	0.4
Lysis	0	0.0	0.0	4	0.0	0.2
Metal-related pathology	0	0.0	0.0	5	0.0	0.3
Heterotopic bone	0	0.0	0.0	2	0.0	0.1
Tumour	0	0.0	0.0	0	0.0	0.0
Implant breakage acetabular	0	0.0	0.0	2	0.0	0.1
Wear acetabular insert	1	0.1	2.6	1	0.0	0.1
Wear head	0	0.0	0.0	1	0.0	0.1
Other	0	0.0	0.0	28	0.0	1.7
Revision, n	38	3.2	100.0	1,641	2.4	100.0
Primary, n	1,182	N/A	N/A	67,248	N/A	N/A

N/A, not applicable.

A total of 86 revisions for acetabular loosening were recorded among 68,430 primary THAs, comprising two revisions (0.17%) in the LL group and 84 revisions (0.12%) in the SL group. At nine years, CPR was 0.3% (95% CI 0.1 to 1.4) for LLs and 0.2% (95% CI 0.2 to 0.3) for SLs, with overlapping CIs indicating no meaningful difference between groups.

## Discussion

Using AOANJRR data, this study evaluated whether LLs increase the risk of revision compared with SLs. The overall revision rate was 3.21% for LL and 2.44% for SL, with no statistically significant difference. Although LLs were hypothesized to raise acetabular loosening risk through higher bone-implant stress, revision for acetabular loosening (0.17% vs 0.12%) was likewise not significant.

To the best of our knowledge, only one previous study has specifically investigated the effect of lateralized (offset) liners on the risk of revision. Archibeck et al^13^ retrospectively reviewed 1,729 primary THAs, of which 120 used an offset liner. They reported an aseptic loosening rate of 0.12% in the standard-offset group and 4.2% in the extended-offset group at a mean follow-up of two years. Notably, 39.4% of THAs in their standard-offset group and 100% in the offset group used conventional polyethylene (CPE) liners. In contrast, the present study, based on AOANJRR data with a 4.5-year follow-up, demonstrated much lower and comparable revision rates for loosening, 0.12% in the SL group and 0.17% in the LL group. The key difference between the two studies lies in the use of highly cross-linked polyethylene (HXLPE) liners in our cohort.

The markedly lower incidence of loosening observed in our study is likely attributable to the widespread use of HXLPE liners. HXLPE has been shown to exhibit significantly reduced wear rates and lower polyethylene particle generation compared with CPE, thereby reducing the risk of osteolysis and aseptic loosening.^17,18^ Consequently, the absence of an increased loosening or revision risk associated with LLs in our cohort suggests that the higher loosening rate reported by Archibeck et al^13^ may have been related more to wear debris generated from CPE liners than to stress concentration at the bone-implant interface.

Age and sex distribution analysis showed that LLs were more frequently used in younger and male patients. This pattern likely reflects underlying anatomical and biomechanical differences. In younger individuals, the narrower femoral canal often limits the range of available femoral component geometries, reducing the ability to accurately match native femoral offset.^11^ Similarly, male patients typically present with greater native femoral and acetabular offset and larger bone dimensions, creating a greater demand for restoring soft-tissue tension and abductor mechanics.^19^ In such cases, surgeons may compensate by using a higher-offset acetabular liner to restore global offset and improve joint stability.

A notable finding in our cohort was the difference in surgical approach between groups. The posterior approach was used in 71.8% of cases with LLs compared with 47.4% in the SL group, whereas the anterior approach was more common in the SL group (40.0% vs 14.4%). This pattern likely reflects surgeons’ preference to use LLs in cases where additional offset or soft-tissue tensioning is required, particularly when employing the posterior approach, which is associated with a higher risk of dislocation.^20,21^ Despite the greater proportion of posterior approach cases in the LL group, the overall revision and dislocation rates were not increased, suggesting that the use of LLs is safe and effective for optimizing hip stability when the posterior approach is used.

The strengths of this study include its large sample size, use of a nationally validated registry with near-complete capture of primary procedures, and a contemporary cohort restricted to highly cross-linked polyethylene liners, minimizing confounding from historical implant materials. The use of time-to-event analysis with multivariable adjustment further strengthens the robustness of the findings.

However, several limitations must be acknowledged. As a registry-based study, radiological data were unavailable, precluding direct assessment of component positioning, true offset restoration, and impingement. In addition, although the AOANJRR has commenced routine collection of patient-reported outcome measures (PROMs) since 2022, PROM data were not available for the study period analyzed, limiting the ability to assess the functional and symptomatic impact of offset modification achieved with LLs. Consequently, although revision and loosening rates were comparable between groups, the effect of LLs on patient-perceived outcomes cannot be determined. Although multivariable adjustment was performed, unmeasured confounders such as implant design variation, femoral head size, or surgeon experience may have influenced outcomes. Furthermore, the relatively small number of LL cases and corresponding revisions may limit statistical power to detect subtle differences in rare failure modes. The selection of liner type was surgeon-dependent, introducing potential indication bias despite statistical adjustment, and nonoperative complications such as dislocation managed without revision are not captured by the registry. Clinically, these findings provide reassurance that LLs can be used to optimize global offset and soft-tissue tension without increasing the risk of revision or loosening in the mid-term, particularly in younger, male patients and when using the posterior approach. Future studies incorporating radiological assessment and PROMs, along with longer-term follow-up, will be valuable to further define the biomechanical and clinical role of LLs in modern THA.

In conclusion, in this large registry-based study, the use of LLs in primary THA did not increase the risk of revision for any reason or for specific causes such as loosening or acetabular loosening. Although LLs were more frequently used in younger, male patients and in association with the posterior approach, their overall survivorship was comparable with SLs. These findings suggest that LLs can be safely used to optimize hip biomechanics and restore offset without increasing the risk of mechanical failure or revision.

**Take home message**

- This study provides the first large-scale registry evidence demonstrating that the use of lateralized polyethylene liners in primary total hip arthroplasty does not increase the risk of revision or acetabular loosening when highly cross-linked polyethylene liners are used.

- These findings support the safe use of lateralized liners to optimize hip biomechanics, restore global offset, and improve soft-tissue tension without compromising implant survivorship.

## Data Availability

The datasets generated and analyzed during the current study are not publicly available, as they were obtained through a study-specific request to the Australian Orthopaedic Association National Joint Replacement Registry (AOANJRR). While aggregate registry data may be available through AOANJRR annual reports, access to the study-specific dataset is restricted in accordance with AOANJRR data governance policies. Further inquiries may be directed to the corresponding author.
